# Recent advances in microbiological and molecular biological detection techniques of tuberculous meningitis

**DOI:** 10.3389/fmicb.2023.1202752

**Published:** 2023-08-28

**Authors:** Wen-Feng Cao, Er-Ling Leng, Shi-Min Liu, Yong-Liang Zhou, Chao-Qun Luo, Zheng-Bing Xiang, Wen Cai, Wei Rao, Fan Hu, Ping Zhang, An Wen

**Affiliations:** ^1^Department of Neurology, Jiangxi Provincial People’s Hospital (The First Affiliated Hospital of Nanchang Medical College), Nanchang, Jiangxi, China; ^2^Department of neurology, Xiangya Hospital, Central South University, Jiangxi Hospital, National Regional Center for Neurological Diseases, Nanchang, Jiangxi, China; ^3^Department of Pediatrics, Jiangxi Provincial People’s Hospital (The First Affiliated Hospital of Nanchang Medical College), Nanchang, Jiangxi, China

**Keywords:** tuberculous meningitis, *Mycobacterium tuberculosis*, microbiology, molecular biology, research progress

## Abstract

Tuberculous meningitis (TBM) is the most common type of central nervous system tuberculosis (TB) and has the highest mortality and disability rate. Early diagnosis is key to improving the prognosis and survival rate of patients. However, laboratory diagnosis of TBM is often difficult due to its paucibacillary nature and sub optimal sensitivity of conventional microbiology and molecular tools which often fails to detect the pathogen. The gold standard for TBM diagnosis is the presence of MTB in the CSF. The recognised methods for the identification of MTB are acid-fast bacilli (AFB) detected under CSF smear microscopy, MTB cultured in CSF, and MTB detected by polymerase chain reaction (PCR). Currently, many studies consider that all diagnostic techniques for TBM are not perfect, and no single technique is considered simple, fast, cheap, and efficient. A definite diagnosis of TBM is still difficult in current clinical practice. In this review, we summarise the current state of microbiological and molecular biological diagnostics for TBM, the latest advances in research, and discuss the advantages of these techniques, as well as the issues and challenges faced in terms of diagnostic effectiveness, laboratory infrastructure, testing costs, and clinical expertise, for clinicians to select appropriate testing methods.

## 1. Introduction

Tuberculosis (TB) is a chronic infectious disease caused by *Mycobacterium tuberculosis* (MTB) that can affect many organs of the human body and is a serious public health problem. Tuberculous meningitis (TBM) is a subacute or chronic inflammation of the meningeal membrane caused by MTB invasion of the subarachnoid space. TBM is the most serious form of extrapulmonary TB and accounts for approximately 1% of TB cases worldwide (Foppiano Palacios and Saleeb, 2020). It has an average mortality rate of 30–40% (Pormohammad et al., 2019; Foppiano Palacios and Saleeb, 2020); the mortality rate is 10% in the first week, rising to 80% in the fifth week (Thwaites, 2013). Most survivors suffer long-term sequelae and even permanent disabilities (Thwaites et al., 2013).

TBM usually involves the pia mater, arachnoid, cerebral parenchyma, and cerebral vessels. The most common symptoms include headache, fever, night sweats, anorexia, nausea, vomiting, and seizures. A typical neurological examination is usually characterised by neck stiffness, fundus abnormalities, an altered level of consciousness, focal neurological deficits, and cranial nerve palsy (Marais et al., 2010). However, TBM is characterised by occult onset, varying clinical symptoms, and poor specificity of early clinical manifestations, which are similar to cryptococcal meningitis (Vidal et al., 2017) and partially treated bacterial meningitis (Wen et al., 2022); therefore, clinical diagnosis is difficult.

The cerebrospinal fluid (CSF) is colourless, transparent, or yellowish. The protein level is moderately elevated, and the sugar and chloride levels were decreased. Dynamic observation of the changes in the CSF is helpful for assisting diagnosis and evaluation of therapeutic effects (Marais et al., 2010). It is believed that in the acute phase of TBM, the CSF is neutrophilic and undergoes a mixed-cell cytological reaction in the subacute phase. After treatment with effective anti-TB drugs, the white blood cell count in the CSF decreases significantly, and neutrophils decrease gradually and disappear completely in the recovery period (Thwaites et al., 2004). Brain imaging examinations of patients with TBM can show hydrocephalus, meningeal enhancement, tuberculoma, cerebral infarction, and skull base enhancement. In addition, pulmonary TB (miliary TB) can be seen on lung scans (Marais et al., 2010; Wen et al., 2023). Typical blood, CSF, and cranial imaging changes improve the reliability of diagnosis, but cannot be used as a definitive diagnostic basis. In recent years, much progress has been made in the diagnosis of TBM using laboratory confirmation. Therefore, this article reviews the current situation of TBM diagnosis in microbiological and molecular biology and discusses the problems and challenges, providing a reference for clinicians.

## 2. Microbiological diagnostic tests

### 2.1. Smear microscopy

The 130-year-old Ziehl-Neelsen (Z-N) staining is a rapid and affordable diagnostic test widely available. Z-N staining uses microscopy to visualise acid-fast bacilli (AFB) in CSF, and remains the cornerstone of laboratory TBM diagnosis (Mai and Thwaites, 2017; Garg, 2019). However, this test usually requires >10,000 organisms to obtain a positive result. As most cases of CSF specimens are paucibacillary, the TBM diagnostic sensitivity is only approximately 10–20% (Thwaites et al., 2000; Wang and Xie, 2018; Stadelman et al., 2022). In recent years, the processing methods of CSF and the staining and microscopy techniques have improved, resulting in a significant improvement in the smear detection rate in some laboratories (Table 1).

**Table 1:** The diagnostic performance for selected tests for tuberculous meningitis (TBM) performed in CSF specimens.

Diagnostic tests for TBM (references)	Limit of detection (cfu/mL)	Sensitivity (%)	Specificity (%)	Role in current practice in TBM & Key points	
**Ziehl-Neelsen staining**	>10,000	8–20	100	**Used in routine practice**	
				• Rapid test & inexpensive • Simple operation • High specificity	• Poor sensitivity •Exposure to toxic substances •Fail to differentiate between MTB & NTM
Wang and Xie (2018), Garg et al. (2019), Stadelman et al. (2022), and Ssebambulidde et al. (2022)					
**MTB culture**	< 10 (Liquid medium) 10–100 (Solid medium)	50–70	100	**Used in routine practice**	
van Zyl-Smit et al. (2011), Bahr et al. (2019), Ahlawat et al. (2020), and Bhasin et al. (2020)				• High specificity (100% gold standard) • MTB detection & DST •Provide a vibrant pure culture	• Variable and low sensitivity • Time consuming • High requirements (biosafety Level III) on the laboratory
**LAMP**	Not available	43–88	80–100	**Conditional recommendation**	
Nagdev et al. (2011), Modi et al. (2016), Sun W. W. et al. (2017), and Yu et al. (2018)				• Rapid test & inexpensive • No special instruments are needed • High biosecurity •Its results are easy to identify	•Variable sensitivity and specificity • CSF specimens required further evaluation •High dependent on primer design
**Xpert**	~ 110	27–85	94.8–100	**WHO recommended for initial diagnosis**	
Rufai et al. (2017), Sharma et al. (2018), Hernandez et al. (2021), and Wakode et al. (2022)				• Rapid test • High specificity • MTB & RIF resistance detection • Semi-quantification of bacillary load	• Poor sensitivity & expensive •Poor diagnosis in EPTB and HIV+ and children • Unable to differentiate between dead and live bacteria
**Xpert ultra**	~ 10–15	44.19–92.9	93.9–100	**WHO recommended for initial diagnosis**	
Halliday et al. (2019), Wang G. et al. (2019), Cresswell et al. (2020), and Huang et al. (2021)				**Compared with Xpert**	
				• Lower LOD • Higher sensitivity	• Variable sensitivity • Cannot rule out TBM
**mNGS**	Not available	44–88.9	86.7–100	**Not recommended as a first-line diagnosis**	
Wang S. et al. (2019), Yan et al. (2020), and Ramachandran et al. (2022)				• Broad-coverage pathogen • Conducive to precision therapy	•Expensive •Difficult to detect intracellular bacteria/fungi •Prone to cross contamination •Affected by non-pathogenic microorganisms

MTB, *Mycobacterium tuberculosis*; NTM, non-tuberculous mycobacteria; DST, drug sensitive test; EPTB, extra-pulmonary; LOD, limit of detection.

#### 2.1.1. CSF collection modifications

One study showed that 58% TBM sensitivity was achieved by obtaining a large volume of CSF (>6 mL) and analysing for >30 min (Thwaites et al., 2004). However, another study argued that taking ≥6 mL CSF may be dangerous for children and prolonged slide examination time > 30 min is not feasible for busy laboratory technicians (Yasuda et al., 2002; Wilkinson et al., 2017; Heemskerk et al., 2018). Kennedy and Fallon (1979) used multiple lumbar punctures to collect CSF for 52 patients with TBM. The Z-N staining results showed that the AFB detection rate was 37% after one lumbar puncture and 83% after four. However, this requires repeated lumbar puncture, which increases the burden on physicians and potential risk of complications, and is usually unacceptable to the patient. Another study showed that sensitivity decreases significantly with prolonged treatment time (Thwaites et al., 2004).

#### 2.1.2. Modified Z-N staining of CSF (triton processing)

Recently, Chen et al. modified the conventional Z-N staining by adopting two additional key steps: centrifugation and separation of CSF samples by cytospin (1,000 ×*g* for 5–10 min) and permeabilisation by Triton X-100 (0.3% for 30 min) (Chen et al., 2012). A pilot study by Chen et al. included 29 patients with culture-confirmed TBM (0.5–1 mL CSF was required); the modified Z-N staining was positive in all patients, while conventional Z-N staining was only 22.9% (Chen et al., 2012). Subsequently, the method was assessed in a larger study of 280 individuals clinically diagnosed with TBM. In this study, the sensitivity of the modified Z-N staining was significantly higher than that of conventional Z-N staining (82.9% vs. 3.3%, respectively), using the clinical diagnosis as the gold standard (Feng et al., 2014). This modified technique significantly improved the detection rate of extracellular mycobacteria and enhanced the intracellular staining of bacilli. In addition, these simple modifications can be applied in most laboratories.

In 2018, the Thwaites team conducted a prospective, international, multicentre study involving 618 individuals with suspected TBM from Vietnam, South Africa, and Indonesia. Conventional and modified Z-N staining with cytospin methods were compared (Heemskerk et al., 2018). Unexpectedly, the sensitivities of the two methods were 66.4 and 67.5%, respectively, when using culture as the reference. This shows that the modified Z-N staining still lacks sensitivity and could not improve the diagnostic performance of TBM. The results of this study are inconsistent with those reported by a Chinese team. Further studies are needed to confirm the performance of the modified Z-N staining for TBM diagnosis.

#### 2.1.3. Microscopy technique improvements

A meta-analysis found that fluorescence microscopy not only improves the sensitivity of sputum smear analysis compared with conventional light microscopy, but also enables slide evaluation in the same microscopic field with a higher efficiency and lower workload (Steingart et al., 2006). However, in addition to needing experienced technicians (Wilkinson et al., 2017; Garg et al., 2018), fluorescence microscopy often requires the use of toxic and carcinogenic dyes, such as auramine-rhodamine or acridine, which are relatively expensive and quench fluorescence quickly (Zou et al., 2016).

A study from China compared the practicality of conventional and modified Z-N staining for CSF specimens and found that the combination of modified Z-N staining and auramine O-free fluorescence microscopy showed significantly higher sensitivity (sensitivity of 96.2% and specificity of 89.3%) (Zou et al., 2016). Compared with light microscopy, the examination time with fluorescence microscopy was halved and the sensitivity increased by 10%, which was supported by the studies of Xia et al. (2013) and Steingart et al. (2006). Moreover, this method reduces harm to technicians and can be performed in a general laboratory.

Light-emitting diode (LED) fluorescence microscopy is cheaper than conventional fluorescence microscopy, using the culture method as a reference standard. Its sensitivity for diagnosing TB is 84%, with a specificity of 98%, a 6% increase in sensitivity compared to traditional Z-N microscopy (Marais et al., 2008; WHO Guidelines Approved by the Guidelines Review Committee, 2011). To compare the performances of the two microscopies in terms of examination time, fading of fluorescently stained slides, and average unit cost, Xia et al. examined 11,276 slides from peripheral laboratories in China. LED fluorescence microscopy was found to have a higher smear-positive detection rate, shorter test time, and lower unit test costs. LED fluorescence microscopy may be an alternative to Z-N as a cost-effective method for detecting MTB (Xia et al., 2013). A review suggested that LED is inexpensive and has many advantages over conventional Z-N-based bright-field microscopy, and has become increasingly popular and is widely used as an alternative light source for fluorescence microscopy in recent years (Ojha et al., 2020).

These studies underscore that the differences in laboratory operator expertise, volume of CSF used, and time spent per sample can vary greatly and significantly affect results. Generally, the diagnostic efficiency of Z-N staining can be improved by increasing the volume of CSF (>6 mL), prolonging slide examination (>30 min), recruiting experienced technicians, adopting a modified Z-N staining method, and examining multiple specimens. However, in most low-resource clinical settings, the performance of this test remains unsatisfactory (Table 1).

### 2.2. Culture

Generally, the sensitivity of MTB CSF culture is slightly superior to that of the AFB smear (Thwaites et al., 2004). In addition, this method can be used to perform drug sensitivity tests and provide strains for other laboratory tests (such as molecular biological examination and strain identification) in the later stage, which is beneficial for TBM diagnosis and treatment. However, this technique is slow and cumbersome, and the extremely low bacterial load means it has variable and low sensitivities in patients with TBM, which makes diagnosis confirmation difficult (Wong et al., 2016, Ahlawat et al., 2020) (Table 1).

#### 2.2.1. Solid culture

Traditional solid media includes Ogawa and Lowenstein-Jensen (L-J). There are many kinds of drug sensitivity tests, which are convenient to guide the selection of clinical anti-tuberculosis drugs and are widely used in developing countries. However, this method is time-consuming; under optimal conditions it takes up to 2–3 weeks to generate results and 3–4 weeks to show drug susceptibility (Caviedes et al., 2000). In addition, L-J culture has approximately 50–70% sensitivity compared to the case definitions (Thwaites et al., 2004).

#### 2.2.2. Liquid culture

Owing to the rapid growth rate of MTB and the shortened drug sensitivity test time, liquid culture is currently superior to solid culture and has gradually replaced traditional L-J culture (Cheng et al., 1994; Caviedes et al., 2000). At present, BACTEC, mycobacteria growth indicator tubes (MGITs), and microscopic observation broth drug susceptibility (MODS) methods are widely used.

##### 2.2.2.1. BACTEC culture system

In a study from India (Baveja et al., 2009), the CSF of 100 children with TBM was tested using BACTEC culture, Z-N staining, L-J culture, and polymerase chain reaction (PCR). The results showed that 22 cases were positive using BACTEC culture and PCR; the coincidence rate between BACTEC culture and PCR for TBM diagnosis was 100%. Among the 22 cases, only two were positive using Z-N staining and six using L-J culture. In another study examining 2,325 CSF samples, the positive rate of the BACTEC 460 TB culture system was 61%, while that of L-J culture was only 7% (Venkataswamy et al., 2007). These results indicate that BACTEC culture has a better sensitivity than traditional L-J culture for TBM.

##### 2.2.2.2. Mycobacteria growth indicator tubes

Compared with L-J culture, MGITs are slightly more sensitive and the detection time is usually 2 weeks (Thwaites et al., 2004; Ssebambulidde et al., 2022). As a liquid culture, it has been indicated that the MGIT 960 has a high specificity in diagnosing TBM, but its sensitivity is only 30–60% (Bhasin et al., 2020). Richard et al. analysed the detection thresholds of four mycobacterium load determination techniques, and the results showed that the limit of detection (LOD) of MGIT was approximately 10 colony forming units (CFU)/mL (1 CFU = 1 organism), L-J culture was 10–100 CFU/mL, and the PCR method Xpert-MTB/RIF was approximately 100 CFU/mL (van Zyl-Smit et al., 2011).

##### 2.2.2.3. Microscopic observation drug susceptibility

The MODS assay, established by Caviedes et al., is a drug susceptibility test based on the liquid culture method by microscopic observation. This assay is inexpensive, reliable, and can be used to simultaneously culture MTB and detect drug susceptibility in a short time (Caviedes et al., 2000). Based on Caws et al. (2007) compared the practicality of MODS to other techniques in the diagnosis of 156 patients with clinically suspected TBM. The positivity rates were 64.9% by MODS, 70.2% by MGIT, 70.2% by L-J culture, and 52.6% by Z-N staining. The MODS assay had low costs ($0.53 per specimen), and the median time to a positive test was significantly reduced to 10 days (Caws et al., 2007). Another study by Sanogo et al. found that the median time for MODS testing was 8 days (Sanogo et al., 2017).

MODS-Wayne is a convenient and sensitive test developed in recent years that can assess pyrazinamide (PZA) resistance by rapidly detecting pyrazinoic acid (a hydrolysed product of PZA), with a sensitivity of 92.7% and specificity of 99.3%. It is a very promising assay and there will be a large market in countries with a high TB burden and limited resources (Alcántara et al., 2019).

##### 2.2.2.4. Other liquid culture methods

To shorten culture time, Bhattacharya et al. developed a novel bilayered medium and compared it with L-J, Middlebrook 7H10 medium, and Kirchner’s medium. After 7 days of culture, the isolation rate of MTB by the bilayered medium was 81.7%, and the earliest positive time was 48 h. The isolation rate of this assay was significantly increased compared to the other methods tested, and the positive detection time was shortened (Bhattacharya et al., 2009). Another study further improved the sensitivity by using a 0.45 μm sterile filter to filter CSF and then culture the membrane (Kumar et al., 2008).

Researchers centrifuged and filtrated CSF from 112 children. Among them, 11 CSF samples were culture-positive after the centrifugation, and 13 CSF samples were culture-positive after filtration; among the samples that were treated with these two methods simultaneously, 17 (15.2%) were culture-positive (Selvakumar et al., 1996). This method improved the culture rate. CSF filtration is simple and inexpensive and can be performed even in hospitals with poor medical conditions. The filtered membranes can be transported to a laboratory with conditions for cultivating MTB.

Compared with the traditional solid culture, the liquid culture system significantly increases the sensitivity, shortens the positive reporting time, and is simple to perform. However, CSF culture is often time-consuming and it is impossible to provide information for clinical diagnosis during the acute phase of TBM. In practice, most patients with TBM have complex and dangerous conditions and cannot wait for culture results before treatment. To ensure the safety of the laboratory environment, the culture of MTB should be performed in a biosafety Level III laboratory, which indirectly increases the cost of testing samples. Nevertheless, when the culture is positive, it plays an important role in subsequent drug susceptibility tests (Table 1).

## 3. Nucleic acid amplification tests

As there are few bacteria in the CSF with TBM, the application of microbial methods such as AFB staining and culture is limited, while molecular biological techniques are advantageous. The molecular detection of pathogens in CSF helps to diagnose TB and anti-TB drug resistance by detecting the MTB genome through NAATs. These are significantly more sensitive than smear assays and take less time diagnose than culture methods (Pormohammad et al., 2019). Furthermore, NAATs can detect TB DNA even after short-term anti-TB treatment (5–15 day) (Thwaites et al., 2004).

In 2019, a systematic review and meta-analysis of NAATs showed that the pooled sensitivity and specificity of NAATs were 82 and 99%, respectively, compared with the reference standard of culture. Compared with clinical case definitions, the sensitivity and specificity of NAAT were 68 and 98%, respectively (Pormohammad et al., 2019) (Table 1).

### 3.1. Multiplex polymerase chain reaction

Multiplex PCR can simultaneously amplify multiple target DNA fragments in a single test. It has been suggested that multiplex PCR can help distinguish MTB from non-tuberculous mycobacterial (NTM) species (Kumar et al., 2014). Kusum et al. used multiplex PCR with three primers, MPB64, IS6110, and protein b, in 210 CSF specimens. The sensitivity and specificity of TBM reached 94.4 and 100%, respectively (Kusum et al., 2011). In addition, researchers from Nepal used IS6110 and MPB64 as primers to diagnose TBM, with a sensitivity of 91.8% and specificity of 100% (Lekhak et al., 2016).

### 3.2. Nested PCR

Nested PCR uses two pairs of PCR primers to amplify a fragment of a target gene, which significantly increases the number of cycles compared to conventional PCR, allowing for easier detection and increased sensitivity (Rios-Sarabia et al., 2016; Garg, 2019). Huang et al. evaluated the efficacy of *rpo*B nested PCR, and the detection of MTB in CSF reached a sensitivity of 86% and specificity of 100% (Huang et al., 2009). Nora et al. obtained a sensitivity of 98% and specificity of 92% using nested PCR in TBM cases (Rios-Sarabia et al., 2016).

In recent years, Chinese researchers have developed a one-tube nested PCR-lateral flow strip test (OTNPCR-LFST) technique, which is fast, sensitive, and simple to operate. It maintains the sensitivity of traditional two-step nested PCR and reduces the chance of cross-contamination and the analysis time. The modification yielded a sensitivity of 89% and a specificity of 100% (Sun Y. et al., 2017).

Real-time PCR (RT-PCR) has been widely used because of quantitative detection, simple operation, small errors, and high automation. It can provide results within 2 h after the DNA extraction process (Ahlawat et al., 2020). Sharma et al. reported that RT-PCR using *rpo*B and MPB64 as targets simultaneously detected drug resistance and MTB in TBM, with a sensitivity of 83.63%, and rifampicin resistance was detected in three out of 110 TBM cases. The results were better than the 1.8 and 10.9% detection rates of smears and cultures, respectively (Sharma et al., 2015). Quantitative real-time PCR (qRT-PCR) was used to detect CSF “filtrates” and “sediments” to diagnose TBM with a sensitivity of 87.6 and 53.1%, respectively. Similar results were obtained using gel-based devR-and IS6110-PCR, suggesting that using filtrates instead of sediments provided more reliable results (Haldar et al., 2009). Additionally, other studies have indicated that the average sensitivity of RT-PCR for TBM diagnosis is 86%, which is superior to that of conventional PCR assays targeting the IS6110 and devR genes (Paverd, 1988; de Almeida et al., 2019; Ahlawat et al., 2020).

### 3.3. Loop-mediated isothermal amplification

Developed in 2000, LAMP is mainly used for DNA amplification of MTB genes IS6110, MPB64, and HspX. A meta-analysis by Chinese researchers showed that LAMP had a pooled sensitivity of 77% and specificity of 99% in extrapulmonary TB (Yu et al., 2018). A multitargeted LAMP assay has also been used for CSF samples. A study from India involving 250 patients (150 patients with TBM) yielded a pooled sensitivity of 88% and specificity of 100% for diagnosing TBM (Modi et al., 2016). In contrast, a study from China showed a sensitivity of 43.0% and specificity of 92.9% for diagnosing TBM using IS6110-based LAMP (Sun Y. et al., 2017). Nagdev et al. retrospectively evaluated the diagnostic value of LAMP in 27 CSF specimens (17 patients with TBM) and this assay yielded a sensitivity of 88% and a specificity of 80% (Nagdev et al., 2011). In one study, IS6110 loop-mediated isothermal amplification had a sensitivity of 88.23% and specificity of 80%, whereas nested PCR had a sensitivity of 52.9% and specificity of 90% (Sun W. W. et al., 2017).

This method does not require sophisticated laboratory infrastructure or strict conditions, can achieve specific and efficient amplification of target DNA at a constant temperature, and results can be read directly with the naked eye. In addition, it is a simple method with high biosecurity. Therefore, it is better suited for widespread implementation in resource-poor, TB-endemic countries (Seki et al., 2018). Currently, LAMP is used in pulmonary TB, and its diagnostic efficacy in extrapulmonary TB is still unclear. It is unclear whether LAMP can be a rule-out test for TB diagnosis in the Xpert MTB/RIF and the new Xpert MTB/RIF Ultra era (Modi et al., 2016). Accuracy is highly dependent on primer design; therefore, its clinical application value needs to be further evaluated (Tables 1, 2).

**Table 2:** Comparison of various molecular biological detection tests for the diagnosis of tuberculous meningitis (TBM).

Tests	Technique Used	Testing purpose	Target gene	Report time (h)	Sensitivity (%)	Specificity (%)	References
LAMP	Thermostatic amplification technique	Etiology	IS6110, MPB64 and HspX etc.	1	43–88	80–100	Nagdev et al. (2011), Modi et al. (2016), and Sun W. W. et al. (2017)
Xpert MTB/RIF	Real-time fluorescence quantitative PCR	Etiology and drug resistance	rpoB	2	27–85	94.8–100	Rufai et al. (2017), Hernandez et al. (2021), and Wakode et al. (2022)
Xpert MTB/RIF Ultra	High resolution melting	Etiology and drug resistance	rpoB, IS6110, IS1081	1.5	44.19–92.9	93.9–100	Wang G. et al. (2019), Cresswell et al. (2020), and Huang et al. (2021)
mNGS	Gene sequencing	Etiology	Standard sequences in the database	24–48	44–88.9	86.7–100	Zhou et al. (2019), Yan et al. (2020), and Ramachandran et al. (2022)
GenoType MTB DRplus	Molecular line probe assay	Etiology and drug resistance	rpoB, inhA, and katG	24	33–76.3	98	Gupta et al. (2015), Solomons et al. (2015), and Mitha et al. (2020)

### 3.4. Xpert MTB/RIF (Xpert)

Xpert is a real-time, fully automated, hemi-nested PCR assay developed by Cepheid (United States). Using *rpoB* as the target gene, Xpert can directly detect the MTB bacilli complex and RIF resistance in CSF and other clinical specimens simultaneously (Committee, WHO Guidelines Approved by the Guidelines Review, 2013; Kay et al., 2022). Xpert was endorsed by the World Health Organization (WHO) for the diagnosis of TB and has been widely used to detect MTB in sputum samples since 2010. In 2013, its utility was further expanded, and it was recommended for all forms of extrapulmonary TB, including TBM (Committee, WHO Guidelines Approved by the Guidelines Review, 2013, 2014).

However, studies have reported that Xpert’s diagnostic performance for TBM is suboptimal (Patel et al., 2023), with a pooled sensitivity of 24–86% and a pooled specificity of 94.8–98.6% against microbially confirmed cases (Boyles and Thwaites, 2015; Rufai et al., 2017; Dorman et al., 2018; Sharma et al., 2018). Two studies from South Africa suggested that increasing the CSF volume tested can increase the diagnostic sensitivity (Patel et al., 2013; Bahr et al., 2015). Similarly, recent studies have shown that testing a large volume (at least 8–10 mL) of centrifuged CSF improves the performance of TBM diagnosis. However, this large volume is not always available in routine testing, especially in children and patients with low cranial pressure (Nhu et al., 2014; Bahr et al., 2016; Khonga and Nicol, 2018). Many studies have also shown that Xpert is highly effective in diagnosing MTB in CSF samples. Results are quick, with a turn-around time within 2 h compared to 2–3 weeks with culture methods (Kohli et al., 2018; Cresswell et al., 2020). In this regard, as a clinical reference standard, culture alone is not sufficient for the confirmation of TBM.

Xpert is rapid and highly automated, with higher biosafety than smear microscopy and lower cross-contamination risk than culture (Cresswell et al., 2018; Dorman et al., 2018; Shapiro et al., 2021). Although Xpert has high specificity for diagnosing TBM, its sensitivity is relatively low in clinical practice (especially in HIV-associated and smear-negative TB patients). While sensitivity can be improved by centrifuging CSF samples, it is necessary to combine it with other highly sensitive detection techniques to improve the early TBM diagnosis (Chen et al., 2022, Patel et al., 2023) (Tables 1, 2).

### 3.5. Xpert MTB/RIF ultra (Xpert ultra)

To reduce the limitations, Cepheid has developed a next-generation Xpert assay called Xpert Ultra, which uses the original reaction system and equipment while doubling the specimen volume. The detection technique was changed from semi-nested to nested PCR. At the same time, two DNA targets (IS6110 and IS1081) were added for more accurate identification of MTB and RIF resistance (Bahr et al., 2018, 2019; Kay et al., 2020). Some studies have confirmed that the LOD is lower for Xpert Ultra (16 cfu/mL) compared with Xpert (114 cfu/mL) (Chakravorty et al., 2017; Committee, WHO Guidelines Approved by the Guidelines Review, 2020). In theory, this means that Xpert Ultra can detect MTB in clinical specimens that contain a small amount of MTB, yielding more positive results (Kohli et al., 2021).

Multiple studies have demonstrated the efficacy of this technique in TBM diagnosis. In 2018, Bahr et al. evaluated the diagnostic value of Xpert Ultra in 23 HIV-positive patients with definite and probable TBM. Xpert Ultra had a 70% sensitivity compared to 43% with MGIT or 43% with Xpert (Bahr et al., 2018). In 2019, Xpert Ultra was shown to be more sensitive than Xpert (44.2% vs. 18.6%, respectively) in 43 HIV-negative patients clinically diagnosed with TBM (Wang G. et al., 2019). Similar results were also obtained in a Chinese study where Xpert Ultra sensitivity was higher than that of Xpert (45% vs. 28%, respectively) in 76 HIV-negative clinical patients diagnosed with TBM (Huang et al., 2021). In 2020, Cresswell et al. further confirmed that the sensitivity of Xpert Ultra was higher than that of Xpert and MGIT in a prospective study that enrolled TBM patients with HIV (Cresswell et al., 2020).

Despite the higher sensitivity of Xpert Ultra, its negative predictive value is inadequate, varying from 61.1–92.7%; a negative result cannot exclude TBM. Therefore, further MGIT 960 culture is recommended for Xpert Ultra negative samples; it is a rule in testing, but not a confidently rule-out test (Cresswell et al., 2020; Donovan et al., 2020a,b). Other studies have noted that Xpert Ultra’s performance is characterised by low LOD and detection of dead bacteria after starting anti-TB or HIV treatment. Therefore, it can detect MTB in cases missed by culture or Xpert (Bahr et al., 2018; Khonga and Nicol, 2018). Although the assay thresholds for both Xpert Ultra and culture were similar, the former had a small number of false-positive results in patients with a history of TB, which could be due to the presence of inactive bacilli or MTB DNA in clinical samples (Arend and van Soolingen, 2018; Dorman et al., 2018; Opota et al., 2019).

Recent research has shown that Xpert Ultra is a potential gamechanger with more advantages than Xpert in TBM diagnosis. Xpert Ultra has increased sensitivity (including accuracy of RIF resistance testing), and detection of MTB in smear-negative, paucibacillary, paediatric, CSF, and other specimens of extrapulmonary TB (Kaswala et al., 2022; Signorino et al., 2022; Sun et al., 2022). In 2017, WHO recommended the use of Xpert Ultra to replace Xpert in diagnosing TBM (Bahr et al., 2018; Rindi, 2022). Despite this, a larger sample size (especially in HIV-negative patients) or more prospective studies are needed to evaluate its diagnostic performance (Tables 1, 2).

### 3.6. Metagenomic next-generation sequencing

mNGS does not rely on traditional microbial culture and can conduct high-throughput sequencing of nucleic acids in clinical samples, rapidly detecting pathogens at the genome level. Theoretically, it can detect all pathogens (viruses, fungi, bacteria, and parasites), providing exact diagnosis for difficult diseases and rare pathogen infections when other testing techniques fail. The development of mNGS may provide a new method for the diagnosis of TBM.

A retrospective study in 2019 reported that the sensitivity of mNGS was 66.7%, which was significantly higher than that of traditional methods such as smear (33.3%), PCR (25.0%), and culture (8.3%), using definite TBM as the reference standard (Wang S. et al., 2019). Analysis by Yan et al. showed the sensitivity of mNGS for TBM in 51 inpatient cases (45 patients with TBM) was significantly higher than that of AFB, MGIT960, MTB PCR, and Xpert (84.4, 0, 22.2, 24.4 and 40.0%, respectively) (Yan et al., 2020). Another retrospective, multi-centre study from China had similar results, indicating that mNGS combined with modified Z-N staining or Xpert could improve TBM diagnosis sensitivity (Chen et al., 2022). A meta-analysis from China evaluating the TBM diagnostic accuracy of mNGS found that mNGS sensitivity was moderate, whereas the specificity was high. Due to the high heterogeneity of the included literature, the conclusions should be treated with caution (Yu et al., 2020). A large prospective study from Uganda involving 368 HIV-infected adults with subacute meningitis showed a sensitivity of 88.9% and specificity of 86.7% by integrating CSF mNGS with a machine learning classifier (MLC) based on host gene expression (Ramachandran et al., 2022). mNGS requires a large volume of CSF (at least 3–5 mL), relatively long detection time (24–48 h), and high cost, which limits wide clinical application. In terms of pathogen detection, mNGS is advantageous for providing a large amount of information that can be searched for clues. From this perspective, mNGS is undoubtedly an important tool. However, mNGS is characterised by a relatively complex detection process, easy contamination by exogenous nucleic acids, long detection time, high professional requirements for result interpretation, difficulty in determining whether the TBM is resistant, and high detection cost.

As the clinical application time of mNGS is low, the clinical significance of mNGS detection remains unclear. The number of sequences of mNGS detection has diagnostic value in which pathogens are clinically significant, and the types of reliable specimens require further research. In summary, mNGS is not recommended as a first-line detection method. It can be considered when traditional tests fail to provide clear etiological results, affecting the accurate diagnosis and treatment, or in emergency situations, where it can used for detection simultaneously with the conventional method (Tables 1, 2).

### 3.7. Other molecular biological detection techniques

GenoType MTB DRplus is a commercially available molecular line probe assay to assess isoniazid and rifampicin resistance by identifying mutations in the *inhA*, *katG*, and *rpoB* genes in MTB isolates. A multicentre prospective study from India examined MTB isolates in CSF specimens from patients diagnosed with TBM. Using the BACTEC MGIT drug sensitivity test as the gold standard, the sensitivity and specificity of this assay to isoniazid resistance was 93 and 97%, and rifampicin resistance was 80 and 98.8%, respectively (Gupta et al., 2015). A small sample study from South Africa found that GenoType MTB DRplus had a sensitivity of only 33% and specificity of 98% (Solomons et al., 2015). Owing to its low sensitivity, complex operation, and high price, it is not widely used in the clinic (Table 2).

High-resolution melting curve analysis (HRM) is a new gene analysis technique based on the different melting temperatures of single nucleotides and the formation of varying melting curves (Sharma et al., 2017). A study evaluated the role of real-time PCR targeting *rpo*B, IS6110, and MPB64 in diagnosing patients with TBM, followed by HRM curve analysis of rpoB gene amplicons to screen for drug resistance. The sensitivity of RT-PCR was much higher than those of culture and smears (83.63, 10.9, and 1.8%, respectively). *rpo*B HRM analysis revealed that rifampicin resistance was detected in three of 110 TBM cases (3.33%) (Sharma et al., 2015). This study indicates that *rpo*B HRM can rapidly diagnose and screen for drug resistance in patients with TBM within 90 min, which is simple and fast, and is more suitable for clinical promotion (Sharma et al., 2015).

In a study of 46 patients with clinically diagnosed TBM by Li et al., the sensitivity of detecting MTB cell-free DNA (cfDNA) (56.5%) in CSF was significantly higher than that of smear (2.2%), culture (13.0%), and Xpert (23.9%) methods (Li X. et al., 2020). A multi-centre prospective study conducted at three TB-specialised hospitals in China found that the cfDNA technique sensitivity was 93.3%, consistent with Xpert Ultra and higher than that of culture methods (13.3%) (Shao et al., 2020).

Matrix-assisted laser desorption ionization-time of flight mass spectrometry (MALDI-TOF MS) is a rapidly developed bioassay with high-throughput characteristics that can be used to identify TB and non-TB bacteria strains. It has the advantages of high resolution, speed, and accuracy and requires fewer bacteria. A previous study showed that the overall detection sensitivity and specificity of MALDI-TOF MS were 92.2 and 100.0% for rifampicin, 90.9 and 98.6% for isoniazid, 71.4 and 81.2% for ethambutol, and 85.1 and 93.1% for streptomycin. This suggests that MALDI-TOF MS has a good performance in predicting the drug resistance of TB bacteria (Wu et al., 2022). Other studies have shown some limitations to the type of drugs that MALDI-TOF MS screens for TB resistance, and drugs with less than 45% of the relevant resistance gene mutations may reduce the sensitivity of this approach (Shi et al., 2022). This technology has some limitations: expensive testing equipment and high technical requirements difficult its promotion in many TB control institutions.

Researchers have used targeted next-generation sequencing (tNGS) to simultaneously detect MTB in bronchoalveolar lavage fluid from patients with TB and to detect resistance to first-line anti-TB drugs, such as rifampicin, isoniazid, ethambutol, and pyrazinamide. Finally, the MTB detection rate of tNGS was 63.1%, which was significantly higher than that of acid-fast bacilli smears (32.3%), cultures (32.3%), and immunological tests (48.6%). Simultaneously, tNGS showed similar sensitivity to Xpert MTB/RIF, using clinical diagnosis as the reference standard. Of the 130 patients, Xpert identified four RIF resistance cases, and phenotypic drug susceptibility testing (pDST) confirmed one as a false positive. All false positive cases were found to be INH resistant using tNGS and pDST. Using pDST as the reference standard, the sensitivity and specificity of tNGS for detecting RIF resistance were 100%, and the sensitivity and specificity for detecting INH resistance were 80 and 100%, respectively (Wu et al., 2023).

Li et al. used digital PCR (dPCR) to detect MTB in 101 HIV-negative patients with TBM, using IS6110 as the target gene, with a higher sensitivity than that of Xpert (70% vs. 30%, respectively) (Li Z. et al., 2020). Ai et al. used a Clustered regularly interspaced short palindromic repeat (CRISPR)-based assay to detect the CSF of 27 patients clinically diagnosed with TBM, with a sensitivity of 73%, which was significantly higher than that of Xpert (54%) and culture (25%) (Ai et al., 2019).

Drug-resistant TB is increasing, and a 5-year retrospective study from India showed that 17.5% of TBM cases are multidrug-resistant (MDR), defined as resistance to both rifampicin and isoniazid (Nagarathna et al., 2008). The Xpert MTB/XDR automated molecular assay (Xpert XDR) is an Xpert Ultra’s next-generation test that can be used to detect mutations in resistance genes for isoniazid, fluoroquinolones, ethionamide, and second-line injectable drugs (amikacin, kanamycin, and capreomycin). In a prospective multicenter diagnostic accuracy study, Xpert XDR showed a sensitivity of 94, 94, 73, 86, and 61% for isoniazid resistance, fluoroquinolones, amikacin, kanamycin, and capreomycin, respectively, with a specificity of 98–100% for all drugs. Its diagnostic efficacy was comparable to that of linear probe analysis. The results showed that Xpert XDR technology has high diagnostic accuracy, quickly and accurately diagnoses drug-resistant tuberculosis, and provides the best treatment plan (Penn-Nicholson et al., 2022). Although Xpert XDR is a promising diagnostic method, further evaluation of its efficacy in clinical specimens from different sources, including special populations such as people with human immunodeficiency virus and children, is needed. BD MAX MDR-TB is a new automated molecular diagnostic platform for detecting MTB in clinical extrapulmonary and pulmonary specimens and its resistance to rifampicin and isoniazid (Sağıroğlu and Atalay, 2021). The system can simultaneously test up to 24 samples and produce results within 4 h (Hofmann-Thiel et al., 2020). The sensitivity and specificity of this assay for detecting rifampicin resistance in MTB were 90 and 95%, respectively, and those for detecting isoniazid resistance in MTB were 82 and 100%, respectively, compared to the drug sensitivity test (Shah et al., 2020).

Recently, India has introduced the Truenat system, which is more suitable for use in low-income countries with high TB prevalence, with high test efficiency and low test cost. Truenat MTB, MTB Plus, and MTB-RIF Dx assays just-in-time test platforms are available (Chakaya et al., 2021; WHO Guidelines Approved by the Guidelines Review Committee, 2021). Several studies have shown that the overall sensitivities of Truenat MTB and MTB Plus reached 83 and 89%, respectively, and the overall specificities reached 99 and 98%, respectively. The overall sensitivity and specificity of Truenat MTB-RiF Dx for the diagnosis of rifampicin resistance were 93 and 95%, respectively. Its diagnostic performance was similar to those of Xpert and Xpert Ultra (Meaza et al., 2021; Penn-Nicholson et al., 2021; Ngangue et al., 2022; Sharma et al., 2022). Moreover, an Indian study of 148 CSF samples with 76 confirmed, 32 probable, and 40 non-TBM controls found that the Truenat assay performed as well as the Xpert Ultra assay in diagnosing TBM (Sharma et al., 2021). However, it is inferior to the Xpert Ultra in determining rifampicin resistance. Nevertheless, compared with GeneXpert, Truenat MTB requires less specimen volume (from 1 mL for GeneXpert to 0.5 mL for Truenat MTB). The Truenat technology only takes 1 h to complete the entire test process. The advantage of Truenat technology is that the rifampicin resistance is tested only when the specimen test results are positive for TB, which significantly reduces the cost of reagents and testing (Acharya et al., 2020; Meaza et al., 2021).

Results of these studies need to be taken with caution as the sample sizes were small. In the future, more cases from different populations will be required to determine the precise role of these methods in TBM diagnosis.

Recently, the continuous development of molecular biotechnology has become a rapid and effective means to improve the etiological detection rate of MTB and realize early and rapid diagnosis of TB and drug-resistant TB. Conventional NAATs are easy to pollute and cumbersome to operate; usually, only qualitative analysis, medium sensitivity, and non-specific amplification are used, but their international standards are complete, the cost of the required instruments and reagents is low, and PCR products can be recycled for other molecular biology experiments and cannot be completely replaced. Conventional and novel NAATs should be a reasonable choice according to the specific situation and their respective advantages and cannot blindly pursue expensive technology. Our understanding of many novel NAATs is insufficient, and their clinical significance remains unclear. Large-scale prospective multicenter clinical studies are needed to accumulate more data.

## 4. Proteomics

In 2011, Kataria et al. applied CSF proteomics to the study of TBM and found that the expression levels of 11 human and eight mycobacterial proteins changed using 2-dimensional difference gel electrophoresis and mass spectrometry. Further screening indicated that arachidonate 5-lipoxygenase has a potential diagnostic value in TBM (Kataria et al., 2011). Ou et al. found differences between the calcium-binding protein A8 (S100A8) and lipoprotein B-100 in the TBM group compared with cryptococcal meningitis and healthy control groups (Mu et al., 2015). Simultaneously, Mu et al. found that the lipoprotein B-100 is a diagnostic protein marker for TBM (Mu et al., 2015). In 2022, Huang et al. identified eight different proteins, which were further verified using enzyme-linked immunosorbent assay (ELISA), and found that a combination of three biomarkers (APOE, APOAl, and S100A8) was a diagnostic protein marker for TBM (Huang et al., 2022).

However, these studies screened only one or two candidate proteins for western blotting or ELISA verification. The study sample size was small, and the individuals in the control group had only one disease and were mainly healthy. Therefore, expanding the sample size and increasing the number of disease types in the control group is necessary for independent verification of the screened proteins.

## 5. Discussion

TBM is a serious threat to human life, and rapid diagnosis and timely treatment are key factors in determining patient prognosis (Wilkinson et al., 2017). However, the sensitivity of existing laboratory tests is low, and sometimes all test results are negative or only one is positive. Therefore, combining multiple tests is necessary to improve the MTB detection rate. In addition, any diagnosis that considers TBM should be examined for the presence of TB foci outside the central nervous system, particularly in the lungs (Galimi, 2011; Donovan et al., 2020c).

Microbiological examination of the CSF is the traditional TBM diagnostic method. However, as MTB concentration in CSF rarely exceeds 100–1,000 colonies/mL (Davis and Wilkinson, 2020), the results of CSF microbiological detection methods are unsatisfactory. MTB culture in CSF is a conventional method for TBM diagnosis, which plays a decisive role. However, this method is time-consuming. Recently, the detection efficiency of MTB has been improved owing to the wide application of liquid culture medium, but rapid TBM diagnosis in the early stage is still difficult (Mai and Thwaites, 2017). AFB staining of CSF smears is the most widely used detection method. It is simple, rapid, and inexpensive, but the sensitivity is poor, so it is necessary to repeat the test and increase the number of smears to improve MTB detection rate (Kennedy and Fallon, 1979). In recent years, improvements have been made in the CSF collection and treatment methods, and in staining and microscopy techniques, resulting in a significant increase in the positive rate of smears in some laboratories. However, the conclusions are not widely verified, and repeated testing and refining of multi-centre, large sample, randomised controlled clinical trials are needed to guide clinical medical strategies accurately, reliably, and effectively.

In recent years, the continuous development of molecular biological techniques has improved MTB detection from the cellular to molecular level. These detection techniques have the advantages of rapidity, repeatability, and high throughput. They have become a rapid and effective means to improve MTB and drug resistance detection rates (Pormohammad et al., 2019). However, these emerging technologies also have limitations. The requirements for laboratory equipment and trained technical operators are higher. Additionally, false-negative results can occur, and a negative result does not rule out TBM even with Xpert Ultra, which is currently the most sensitive diagnostic tool (Cresswell et al., 2020; Donovan et al., 2020a,b). This emphasises that a clear understanding of these detection techniques is needed by clinicians, otherwise they may cause treatment delays or misdiagnosis, greatly increasing the economic burden on patients.

Research on the microbiology and molecular biology of TBM still faces many challenges. First, the inclusion criteria for patients with TBM are inconsistent, making parallel comparisons difficult. According to the Marais score criteria (in 2010), TBM is classified as definite, probable, and possible (Marais et al., 2010). Some studies included only confirmed TBM, while others included confirmed and probable TBM or all suspected TBM, as well as some studies including people with HIV, making direct comparisons difficult between the few published studies. Second, including CSF specimens in assessing the diagnostic value of the test is difficult. CSF is obtained through an invasive lumbar puncture, and only a limited number of CSF samples can be taken for testing at a time. Due to the relatively low incidence of TBM, many studies have small sample sizes, and the reliability of the diagnostic value assessed is not high. Third, control groups are often inconsistent from study to study; therefore, caution is needed when comparing and evaluating the results. Studies with a greater number of control groups are more difficult, but the conclusions are more reliable. Fourth, molecular biological technologies are more expensive than traditional microbiological tests for MTB, making it difficult for these technologies to be utilised in low-and medium-developed countries where a larger proportion of patients have TBM. In future research, it is particularly important to develop molecular detection technology suitable for regions with limited medical conditions on the basis of comprehensive measurement of technical performance and economic affordability.

## 6. Conclusion

In conclusion, although great progress has been made in the laboratory diagnosis of TBM, challenges remain. With the advent of precision diagnosis and treatment, molecular biology has been widely studied and applied. However, we must emphasize that classical microbiology technology should not be ignored, and the two should be combined to complement each other. Clinicians need to understand the characteristics of various diagnostic methods and choose the most reasonable and economical detection methods according to different patient conditions, instead of blindly seeking new methods and casting nets.

## Author contributions

AW, W-FC, and E-LL designed the study. AW wrote the manuscript. S-ML, Y-LZ, and C-QL did the data searches and study selection. Z-BX and WC did the data synthesis. WR, FH, and PZ created the tables. All authors contributed to the article and approved the submitted version.

## Funding

This work was supported by grant from Jiangxi Provincial Health Commission Foundation (no. 202310122).

## Conflict of interest

The authors declare that the research was conducted in the absence of any commercial or financial relationships that could be construed as a potential conflict of interest.

## Publisher’s note

All claims expressed in this article are solely those of the authors and do not necessarily represent those of their affiliated organizations, or those of the publisher, the editors and the reviewers. Any product that may be evaluated in this article, or claim that may be made by its manufacturer, is not guaranteed or endorsed by the publisher.
